# N_2_O and CH_4_ emission from *Miscanthus* energy crop fields in the infertile Loess Plateau of China

**DOI:** 10.1186/s13068-018-1320-8

**Published:** 2018-12-03

**Authors:** Jia Mi, Wei Liu, Xuhong Zhao, Lifang Kang, Cong Lin, Juan Yan, Tao Sang

**Affiliations:** 1 Key Laboratory of Plant Resources and Beijing Botanical Garden, Institute of Botany, Chinese Academy of Sciences, No. 20 Nanxincun, Xiangshan, Beijing, 100093 China; 2 Institute of Loess Plateau and Shanxi Green Development Research Centre, Shanxi University, Taiyuan, 030006 Shanxi China; 3 University of Chinese Academy of Sciences, Beijing, 100049 China; 4 Key Laboratory of Plant Germplasm Enhancement and Speciality Agriculture, Wuhan Botanical Garden, Chinese Academy of Sciences, Wuhan, 430074 Hubei China; 5 State Key Laboratory of Systematic and Evolutionary Botany, Institute of Botany, Chinese Academy of Sciences, Beijing, 100093 China

**Keywords:** Energy crop, Greenhouse gases, Marginal land, Methane, *Miscanthus lutarioriparius*, Nitrous oxide

## Abstract

**Background:**

The greenhouse gas (GHG) mitigation is one of the most important environmental benefits of using bioenergy replacing fossil fuels. Nitrous oxide (N_2_O) and methane (CH_4_) are important GHGs and have drawn extra attention for their roles in global warming. Although there have been many works of soil emissions of N_2_O and CH_4_ from bioenergy crops in the field scale, GHG emissions in large area of marginal lands are rather sparse and how soil temperature and moisture affect the emission potential remains unknown. Therefore, we sought to estimate the regional GHG emission based on N_2_O and CH_4_ releases from the energy crop fields.

**Results:**

Here we sampled the top soils from two *Miscanthus* fields and incubated them using a short-term laboratory microcosm approach under different conditions of typical soil temperatures and moistures. Based on the emission measurements of N_2_O and CH_4_, we developed a model to estimate annual regional GHG emission of *Miscanthus* production in the infertile Loess Plateau of China. The results showed that the N_2_O emission potential was 0.27 kg N ha^−1^ year^−1^ and clearly lower than that of croplands and grasslands. The CH_4_ uptake potential was 1.06 kg C ha^−1^ year^−1^ and was slightly higher than that of croplands. Integrated with our previous study on the emission of CO_2_, the net greenhouse effect of three major GHGs (N_2_O, CH_4_ and CO_2_) from *Miscanthus* fields was 4.08 t CO_2eq_ ha^−1^ year^−1^ in the Loess Plateau, which was lower than that of croplands, grasslands and shrub lands.

**Conclusions:**

Our study revealed that *Miscanthus* production may hold a great potential for GHG mitigation in the vast infertile land in the Loess Plateau of China and could contribute to the sustainable energy utilization and have positive environmental impact on the region.

**Electronic supplementary material:**

The online version of this article (10.1186/s13068-018-1320-8) contains supplementary material, which is available to authorized users.

## Background

Mitigating greenhouse gas (GHG) emissions is one of the most important environmental benefits of using bioenergy crops replacing fossil fuels [1–4]. It is well known that higher concentrations of CO_2_ may cause significant atmospheric warming through its greenhouse effect with a mean residence time of 5 years. In addition to CO_2_, nitrous oxide (N_2_O) and methane (CH_4_) are considered to be the main GHGs. Each molecule of N_2_O has the potential to contribute 300-fold to the greenhouse effect compared to each molecule of CO_2_ and the mean residence time in the atmosphere is about 120 years [5]. Moreover, N_2_O can cause ozone depletion in the Earth’s stratosphere [6]. Soil emissions from nitrification and denitrification are the largest global sources of N_2_O, especially from the disturbed soils following land use changes. For CH_4_, it has the potential to contribute 25-fold or more to the greenhouse effect relative to each molecule of CO_2_ with the mean residence time of about 9 years [7]. With the CH_4_ content in the atmosphere continuously increasing, there is a growing concern that bioenergy production on commercial farms (e.g., in Europe) could have higher CH_4_ emissions than annual barley or maize lands due to the differences of soil moisture and temperature [8–10].

Although many relevant works of GHG emission from bioenergy production have been published, most of them are focused on the Life Cycle Analyses or GHG emission in the energy crop field scale [11–13]. Regional GHG emission studies are rather sparse and how soil temperature and moisture affect the emission potential in the large scale remains to a large extent unknown. Therefore, it is difficult to provide valuable guidance to policymakers for developing bioenergy [14, 15]. To address this problem, one reasonable approach is to choose the suitable energy crops grown in marginal land and build up a model to bridge the laboratory-based N_2_O and CH_4_ fluxes at the site scale to GHG emissions at the regional scale.

*Miscanthus* has been identified as a promising second-generation energy crop to cultivate due to its low input demand and high biomass productivity, especially its high cellulose content [16, 17]. Previous studies demonstrated that *Miscanthus lutarioriparius*, a C_4_ endemic species in central China, can adapt to the semi-arid regions and produce high feedstock in North China [18]. Furthermore, growing this C_4_ grass in the Loess Plateau has many environmental benefits, including soil and water conservation, carbon sequestration and soil restoration in the infertile and soil-eroded region [19–21]. Thus, bioenergy production in the large scale may bring a sustainable solution to this region [22, 23]. Most of natural vegetation covers in the Loess Plateau, including forests, shrubs, and grasses, have been cleared and lands converted to arable land use following population expansion, even cultivation on steep slopes [24, 25]. Irrational land use has created severe environmental problems, which turned this region into one of the most eroded zones of the world [26]. With recent China’s “Grain to Green Project”, a large number of barren or low-yield cultivated land and wasteland have been used to restore the vegetation and soil of a sustainable ecosystem [27–30], which holds a great potential for the production of *Miscanthus* energy crops [31, 32].

Here we sampled the top soils from two *Miscanthus* fields. One is in Qingyang of the Gansu Province (QG), located in the center of the Loess Plateau as an energy crop domestication site. The other is located in Jiangxia of the Hubei Province (JH) near its native habitats as the control site. Fluxes of N_2_O and CH_4_ were periodically measured using a short-term (28 days) laboratory microcosm incubation approach under different conditions of soil temperature and moisture. In addition, a regional model was developed to link the experimental conditions of temperature and moisture in the laboratory with the soil temperature and precipitation in the field to estimate the annual GHG emission of *Miscanthus* production in the marginal lands across the Loess Plateau.

## Results

### Soil N_2_O and CH_4_ release rates

According to the standard procedures of soil incubation and GHG measurement, the soil N_2_O and CH_4_ release rates were analyzed using repeated measures analysis (Table 1). For N_2_O rates, the results indicated that the effects of soil moisture, incubation time, soil depth and site were significant, but the effect of soil temperature was not. Interestingly, most of the effects of the interaction between two factors were also significant, except the pairs of moisture and site, temperature and soil depth, incubation time and soil depth, and soil depth and site. For the case of no less than three impacted factors, the interactive effects carried by soil moisture, temperature, incubation time and/or site were only significant.

**Table 1:** ANOVA of N_2_O-N and CH_4_-C release rates

Effects	*df*	N_2_O-N		CH_4_-C	
		*F*	*P*	*F*	*P*
Moisture (M)	2	134.54	< 0.0001	12.19	< 0.0001
Temperature (Te)	3	2.21	0.0856	8.21	< 0.0001
Time (Ti)	6	63.81	< 0.0001	9.41	< 0.0001
Depth (D)	1	5.48	0.0194	0.05	0.8240
Site (S)	1	39.88	< 0.0001	34.91	< 0.0001
M × Te	6	3.96	0.0006	4.71	< 0.0001
M × Ti	12	14.73	< 0.0001	1.98	0.0230
M × D	2	11.12	< 0.0001	1.75	0.1740
M × S	2	2.90	0.0554	1.24	0.2890
Te × Ti	18	17.62	< 0.0001	8.66	< 0.0001
Te × D	3	0.33	0.8007	0.66	0.5770
Te × S	3	12.65	< 0.0001	13.37	< 0.0001
Ti × D	6	0.54	0.7761	0.78	0.5830
Ti × S	6	3.98	0.0006	4.40	< 0.0001
D × S	1	0.15	0.6970	0.05	0.8180
M × Te × Ti	36	5.08	< 0.0001	6.27	< 0.0001
M × Te × D	6	0.12	0.9944	1.06	0.3860
M × Te × S	6	3.39	0.0025	4.52	< 0.0001
M × Ti × D	12	0.94	0.5105	0.39	0.9680
M × Ti × S	12	1.74	0.0541	6.52	< 0.0001
M × D × S	2	0.71	0.4884	1.18	0.3070
Te × Ti × D	18	0.35	0.9947	1.06	0.3850
Te × Ti × S	18	3.10	< 0.0001	5.74	< 0.0001
Te × D × S	3	0.39	0.7618	1.34	0.2600
Ti × D × S	6	1.07	0.3810	0.21	0.9730
M × Te × Ti × D	36	0.56	0.9849	0.51	0.9930
M × Te × Ti × S	36	1.79	0.0030	2.97	< 0.0001
M × Te × D × S	6	0.59	0.7395	0.97	0.4420
M × Ti × D × S	12	0.64	0.8129	0.47	0.9320
Te × Ti × D × S	18	0.60	0.9049	0.3030	0.9980
M × Te × Ti × D × S	36	0.59	0.9755	0.3990	0.9990
Error	1680				

The repeated measures analysis for soil CH_4_ release rates showed that soil moisture, temperature, incubation time, and site were all significant, except the soil depth. Most of the two-factor interaction effects were also significant, except the effects of soil moisture and depth, soil moisture and site, incubation temperature and soil depth, incubation time and soil depth, and soil depth and site. The interaction effects of soil moisture, temperature and incubation time (or site) had significant effects on CH_4_ release rates. Similar interaction of factors with significant impacts include the interaction effects of soil moisture (or soil temperature), incubation time and site, and the interaction effects of soil moisture, temperature, incubation time and site.

**Fig. 1:**
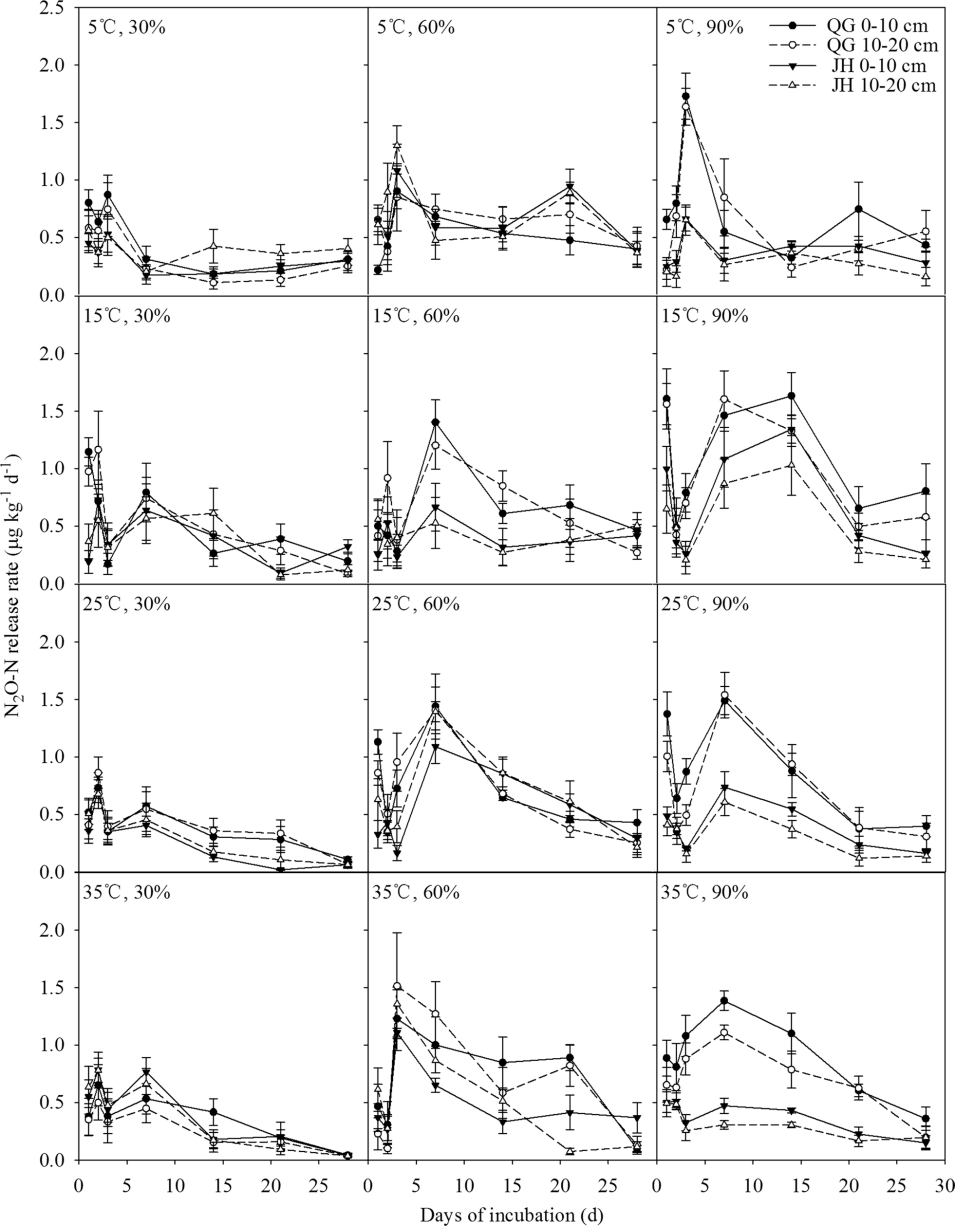
Soil N_2_O-N release rates with days of incubation of soil samples supporting *Miscanthus lutarioriparius* in the two field sites

**Fig. 2:**
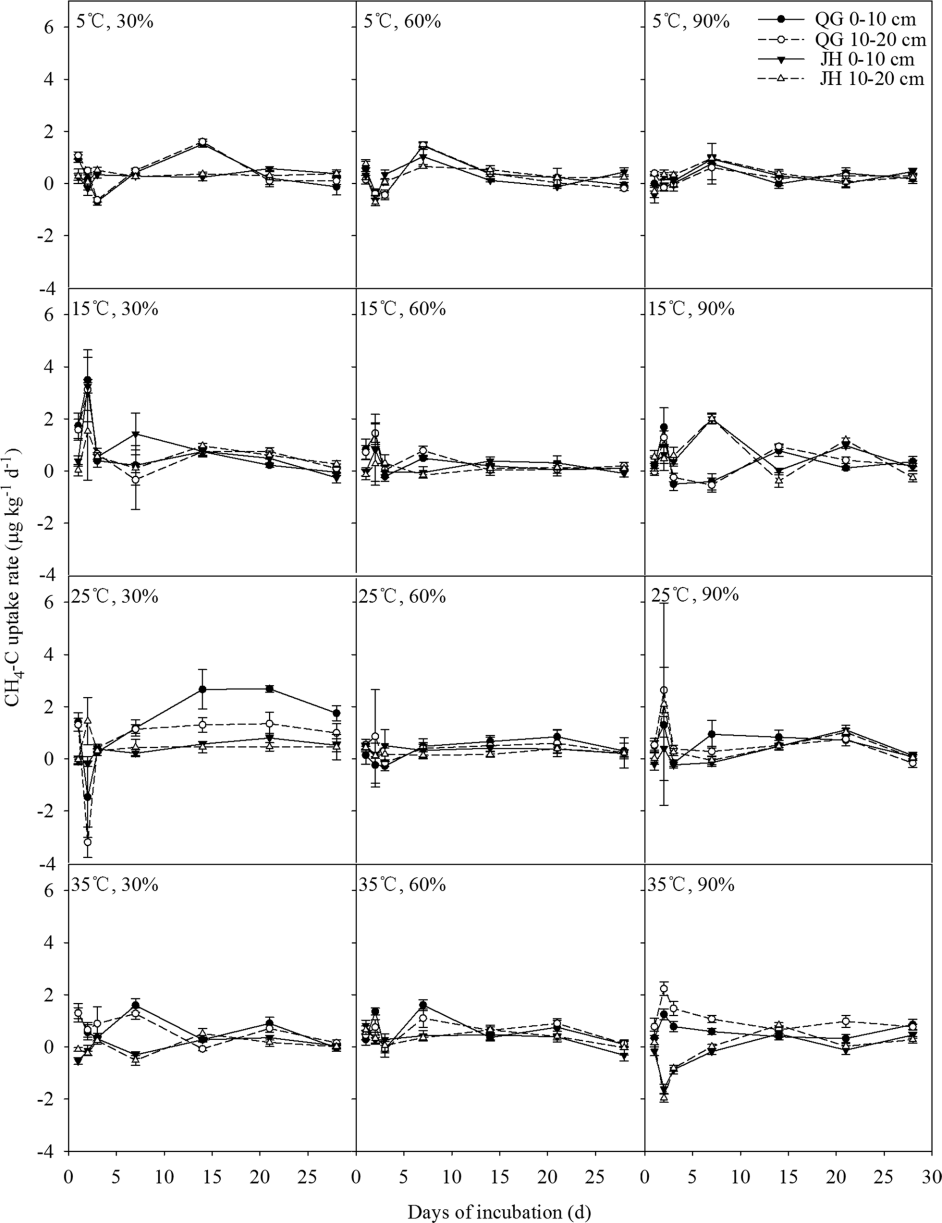
Soil CH_4_-C uptake rates with days of incubation of soil samples supporting *Miscanthus lutarioriparius* in the two field sites

Under the incubation conditions of 5 °C combined with 30%, 60%, and 90% water-filled pore space (WFPS), N_2_O-N release showed similar patterns that the highest release rates were observed on the 3rd day and then rate decreases gradually (Fig. 1). In the condition of 30% WFPS combined with 15 °C, 25 °C and 35 °C soil temperature, N_2_O-N release showed two obvious peak values on the 3rd day and 7th day. Under 60% and 90% WFPS combined with 15 °C, 25 °C and 35 °C soil temperature, a sustained higher release rate appeared from 3 to 14 days. For the CH_4_-C fluxes, they generally showed big fluctuations in absorption and release for the first 7 days, and then showed slow absorption rates in the rest of the incubation period (Fig. 2). Between the soils of two sites, N_2_O-N release rates and CH_4_-C uptake rates decreased with the increase of soil depth. The N_2_O-N average release rate and the CH_4_-C average uptake rate were higher at QG site in the whole 28-day incubation period (Figs. 1, 2). Under the theoretical optimum conditions of 25 °C and 60% WFPS, the maximum release rate of N_2_O-N in QG was 1.44 μg kg^−1^ day^−1^ in the 0–10 cm soil layer and in JH, 1.39 μg kg^−1^ day^−1^ in the 10–20 cm soil layer. Under the same incubation conditions, the maximum CH_4_-C uptake rate in QG was 0.86 μg kg^−1^ day^−1^ in the 10–20 cm soil layer and 0.51 μg kg^−1^ day^−1^ in the 0–10 cm soil layer in JH, respectively.

### Variation of cumulative N_2_O and CH_4_

**Fig. 3:**
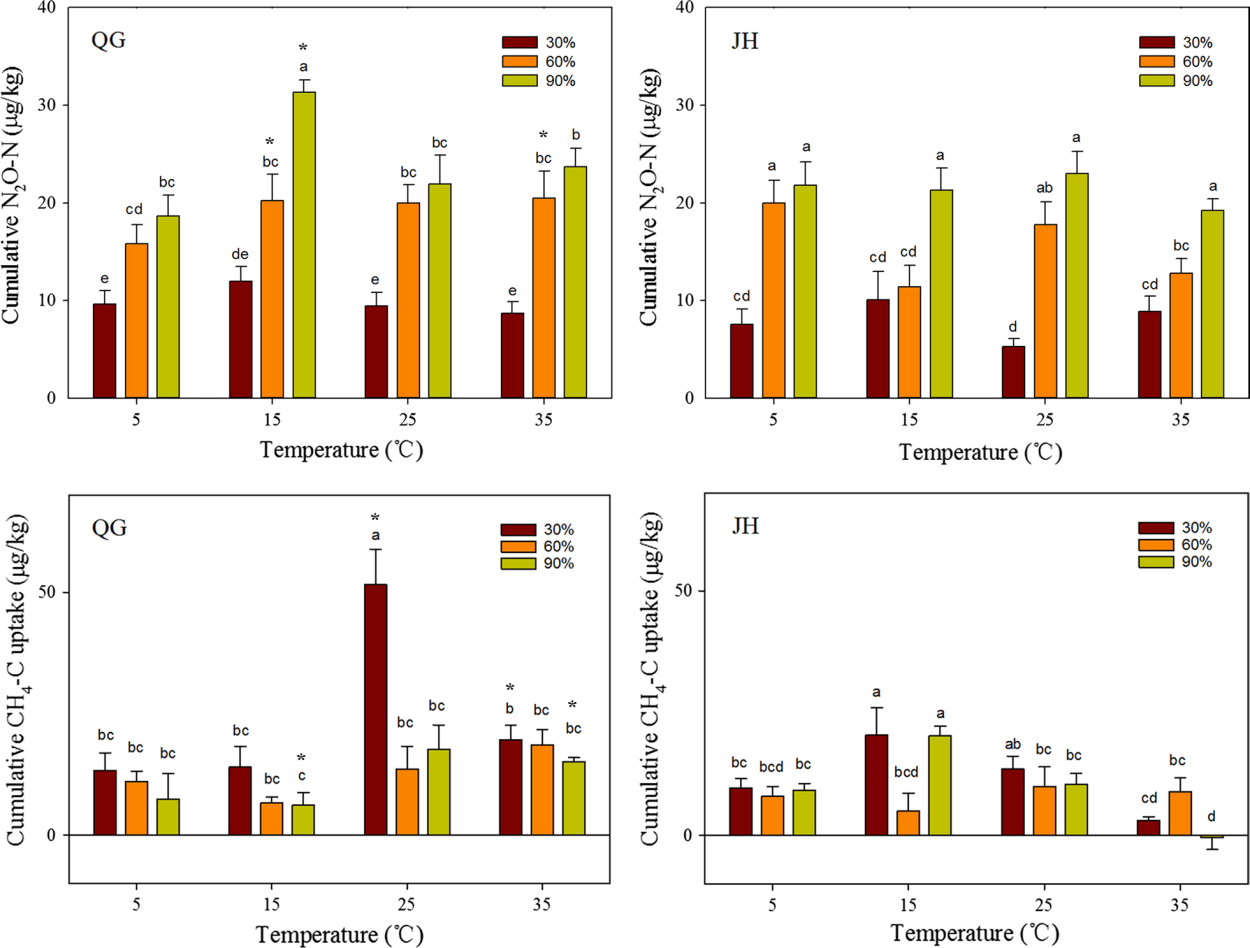
The effects of soil temperature and moisture on cumulative N_2_O-N emission and CH_4_-C uptake of the 0–10 cm soil layer in the sites of QG and JH. The cumulative N_2_O-N and CH_4_-C for each treatment was the average of three replicates (error bars denote standard error of mean). Bars with the same letter were not significantly different in the least significant difference (LSD) tests reported from ANOVA. Asterisk indicates a significant level of difference between QG and JH under same soil temperature and moisture conditions at *P* = 0.05

In both QG and JH, the single-factor effect and interaction effects of soil moisture and temperature on cumulative N_2_O-N were all highly significant (Table 2). The interaction effects of moisture and soil depth were significant in the JH site. For the cumulative CH_4_-C, the soil temperature and interaction effects of temperature and moisture in both QG and JH were highly significant while the effect of soil moisture was significant in the QG site (Table 2). Compared with the results of all treatments for the 28-day incubation, the highest N_2_O-N emission rate was 31.32 μg kg^−1^ at 0–10 cm soil depth of QG (under 15 °C and 90% WFPS), and the highest CH_4_-C uptake rate was 51.54 μg kg^−1^ at the 0–10 cm soil depth of JH site (under 25 °C and 30% WFPS) (Fig. 3).

**Table 2:** Generalized linear mixed model (GLMM) results for the differences of effects on cumulative N_2_O-N emission and CH_4_-C uptake

Effects	*df*	N_2_O-N				CH_4_-C			
		QG		JH		QG		JH	
		*F*	*P*	*F*	*P*	*F*	*P*	*F*	*P*
Moisture	2	74.42	< 0.0001	57.31	< 0.0001	11.63	< 0.0001	3.01	0.0528
Temperature	3	7.54	0.0001	3.44	0.0191	12.87	< 0.0001	10.08	< 0.0001
Depth	1	1.68	0.1981	2.50	0.1168	0.54	0.4644	0.09	0.7669
Moisture × temperature	6	2.32	0.0371	3.93	0.0013	4.89	0.0002	6.94	< 0.0001
Moisture × depth	2	1.04	0.7966	5.29	0.0063	1.97	0.1445	0.56	0.5702
Temperature × depth	3	0.34	0.3576	0.12	0.9460	2.57	0.0578	0.81	0.4905
Moisture × temperature × depth	6	0.32	0.9260	0.30	0.9381	1.21	0.3069	0.17	0.9851
Error	120

In the site of QG, the trend of cumulative N_2_O-N release was first increased and then decreased with the increased incubation temperatures and the highest values appeared at 15 °C under all three moisture conditions (Fig. 3, Additional file 1: Fig. S1). However, the cumulative N_2_O-N releases in JH were more complicated. Under each of the experimental temperature, the values increased with the increase of soil moisture. Under 30% or 90% WFPS, the cumulative values of N_2_O-N at all temperatures showed no significant difference between each other. Under the 60% WFPS, the cumulative values of N_2_O-N at 5 °C and 25 °C showed no significant difference between each other but were higher than those at 15 °C and 35 °C.

In the site of QG, the differences of cumulative CH_4_-C uptake between water treatments were not significant at 5 °C, 15 °C and 35 °C soil temperature, and the significantly higher value occurred at 30% WFPS of soil moisture in the 0–10 cm soil layer (Fig. 3). In the site of JH, the cumulative CH_4_-C uptakes were significantly higher under 15 °C soil temperature and 30% or 90% WFPS treatments. But a slight accumulation of CH_4_-C uptake occurred under the incubation conditions of 35 °C and 90% WFPS. The accumulations of N_2_O-N release at QG site were significantly higher than the ones at JH site under the conditions of 15 °C combined with 60% or 90% WFPS and 35 °C combined with 60% WFPS (Fig. 3). The accumulations of CH_4_-C uptake at QG site were significantly higher than the ones at JH site under the conditions of 25 °C combined with 30% WFPS and 35 °C combined with 30% or 90% WFPS, but were significantly less than the ones at JH site under conditions of 15 °C combined with 90% WFPS (Fig. 3).

### Estimate of GHG emission pattern and the effects in the Loess Plateau

**Fig. 4:**
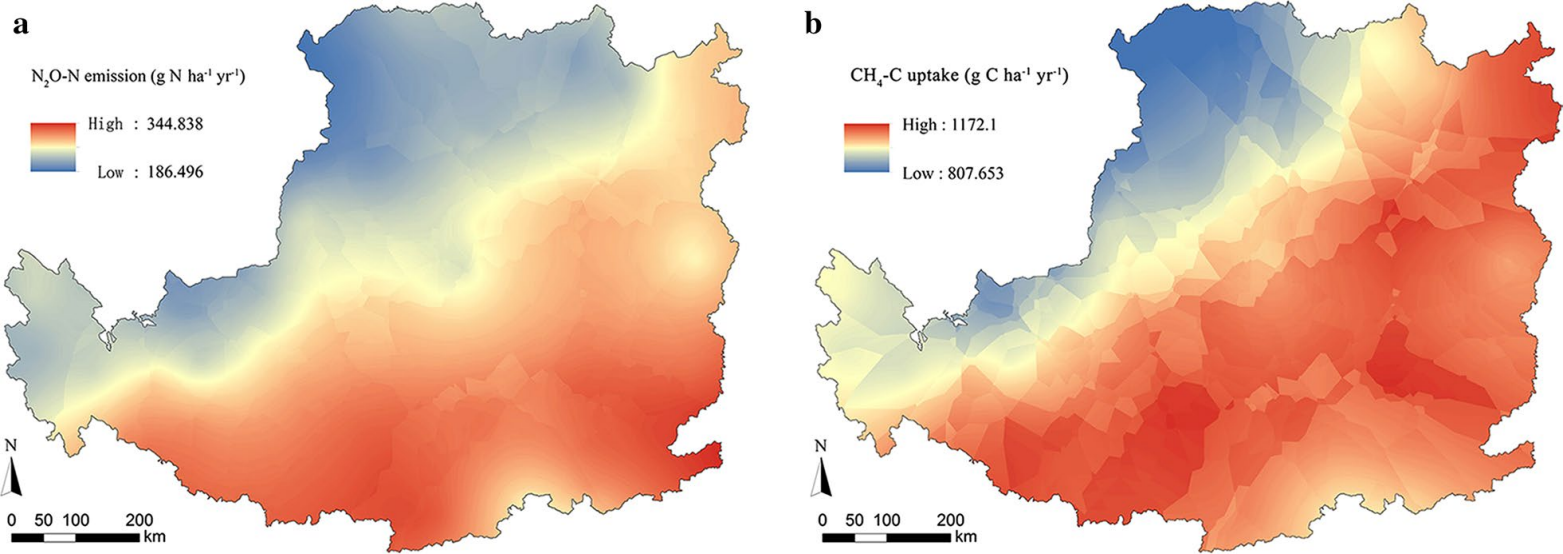
Map of modeled N_2_O-N and CH_4_-C emissions from *M. lutarioriparius* production across the Loess Plateau

**Fig. 5:**
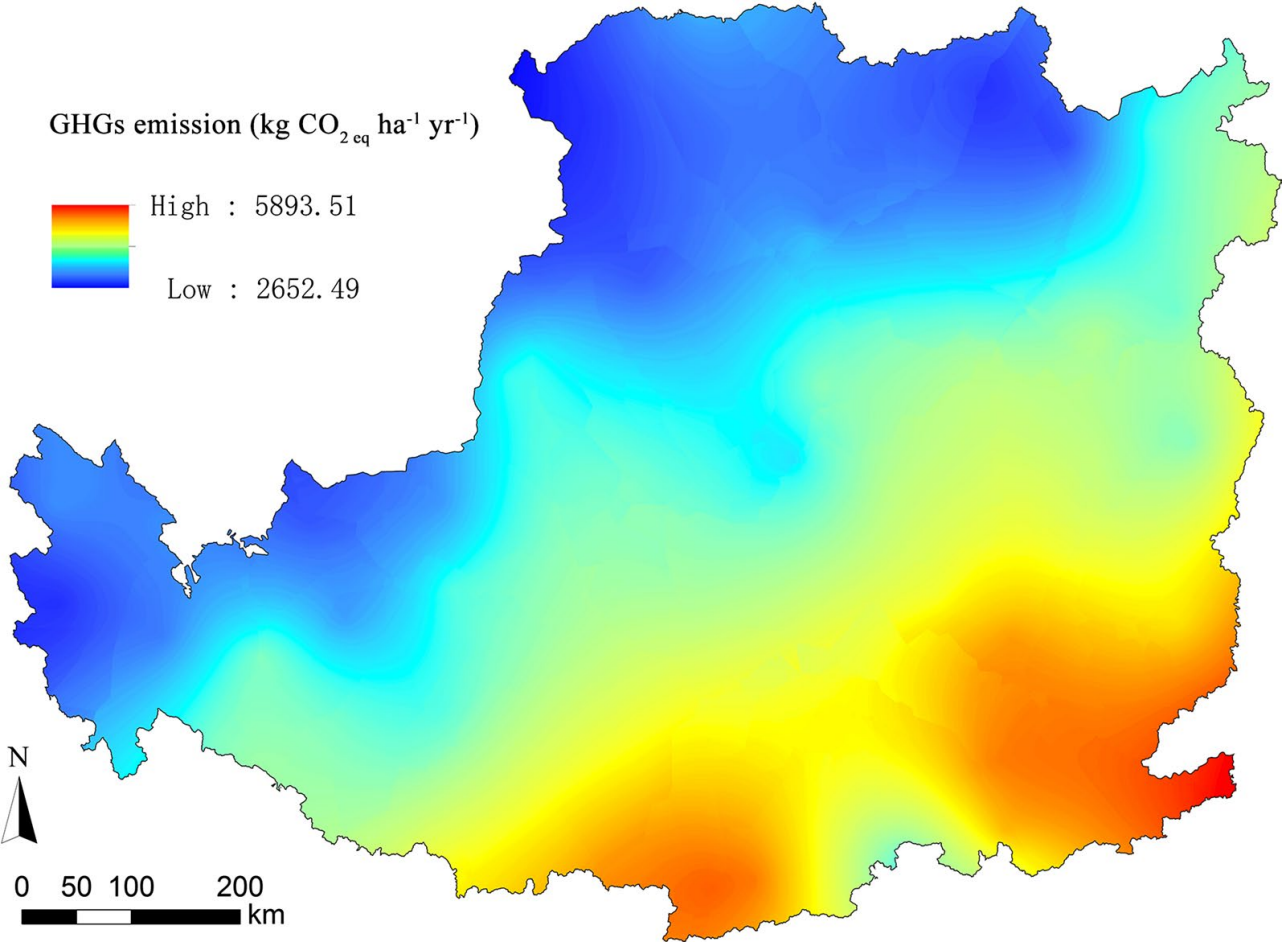
Map of modeled three major GHG emissions (converted into CO_2_ equivalents) from *M. lutarioriparius* production across the Loess Plateau

Based on the release rates of N_2_O and CH_4_ measured in the 28-day laboratory incubation (Additional file 1: Figs. S2, S3), a regional model was built to estimate the average rates of N_2_O and CH_4_ in the whole year under different conditions of temperature and moisture in QG (Additional file 1: Tables S1, S3). Daily N_2_O and CH_4_ release rates were estimated based on the field conditions of temperature and precipitations in the field sites (Additional file 1: Tables S2, S4). When the estimates of soil cumulative N_2_O and CH_4_ (0–20 cm layer) were extended to the whole region of the Loess Plateau, the average release value of N_2_O-N was 0.27 kg ha^−1^ year^−1^ and the average uptake value of CH_4_-C was 1.06 kg ha^−1^ year^−1^. The total amount of N_2_O-N emission was 9.16 million kg year^−1^ in the 33.3 Mha marginal lands available in the Loess Plateau (Fig. 4a) and the amount of CH_4_-C uptake was 35.16 million kg year^−1^ (Fig. 4b). To estimate the influence of N_2_O and CH_4_ on the overall GHG balance of *Miscanthus* production, soil fluxes of N_2_O and CH_4_ have been converted to CO_2_ equivalents. N_2_O emission was 133.96 kg CO_2eq_ ha^−1^ year^−1^ and CH_4_ uptake was 35.19 kg CO_2eq_ ha^−1^ year^−1^. Integrated with our previous study on the emission of CO_2_, major GHG (N_2_O, CH_4_, and CO_2_) emission was 4.08 t CO_2eq_ ha^−1^ year^−1^ and the total GHG amount was 135.97 million t CO_2eq_ year^−1^ in the entire marginal lands available in the Loess Plateau (Fig. 5).

## Discussion

### Soil hydrothermal condition and patterns of N_2_O emission and CH_4_ uptake

The environmental conditions of habitats differed greatly in the original growth and domestication regions of *Miscanthus*. Whether *Miscanthus* introduction to the Loess Plateau could affect soil properties and soil GHG emissions is an important issue that must be clarified in the initiatives for semi-arid marginal land use. Temperature and precipitation are important environmental factors affecting soil GHG release [33]. In most recent studies, the release of greenhouse gases was estimated by the change of atmospheric temperature [7, 34]. However, the simulation process of GHG emissions using only atmospheric temperature factors had the problem of poor accuracy of assessment values [35]. In our study, an assessment model incorporating soil type, soil temperature and soil moisture into the greenhouse gas release was introduced for the Loess Plateau region.

The results suggested that soil temperature had much greater influence on the CH_4_ release than soil moisture or the interactions between moisture and temperature (Table 2). The CH_4_ uptake was accelerated by increasing soil temperature in the two sites. Especially, the tendency increases even more obviously from 15 to 25 °C in QG. The cumulative of CH_4_ release under 30% WFPS reached the highest value at 25 °C among four temperature treatments, and they reached the highest value at 25 °C or 35 °C under 60% and 90% WFPS (Fig. 3 and Additional file 1: Fig. S1). In JH, the cumulative of CH_4_ release under 30% and 90% WFPS reached the highest value at 15 °C. Furthermore, the CH_4_ uptake was gradually reduced with the temperature increase from 15 to 35 °C in the JH site. Due to the limitation of bioactive substance properties regulated by environmental conditions and the combined action of methanotrophs and methanogens, it has been proposed that soil CH_4_ uptake is restrained after the soil temperature reached a threshold (15–25 °C in our study). A previous study showed most types of anaerobic methane bacteria are suitable for medium temperature condition (12–20 °C), and their biological activities drop rapidly at lower or higher temperatures [36].

However, soil moisture had a greater impact on N_2_O emission than soil temperature (Table 2). Our results also supported the earlier reports on the influence of soil N_2_O emission by soil water availability [37]. Soil N_2_O fluxes were lower with low soil moisture, even with the higher soil organic matter (SOM) or soil available nitrogen. In previous studies, the upper threshold is 80–90% WFPS [38]. In our study, however, this threshold did not appear probably because of the specificity of soil properties in tested sites, such as the soil clay content and soil texture. Soil moisture diffusion is quicker with lower proportion of clay content, which does not cause oxygen deficit for the process of nitrification and denitrification mediated by soil microbial metabolism. Compared with the results in JH site, the effect of soil depth on N_2_O release rate appeared significantly in QG, which indicated that the heterogeneity of different soil layers in QG was higher than in JH. The results of soil incubation showed that the potential N_2_O release from soil was reduced under the conditions of drought or high temperature, which meant drought or high temperature was advantageous for the retention of soil nitrogen.

Net nitrification rates in the anoxic soils in the Yangtze River basin were significantly lower than in aerobic soils in the Loess Plateau, which resulted in the lower redox potential and limiting methane absorption [39]. This was the reason why less cumulative CH_4_ uptake was observed in JH site under most of the incubation conditions. Soil moisture diffusion depends partly on the characteristics of soil particles and is quick in low-clay content soils but slow in high-clay content soils, which was probably caused by the alleviated hypoxia for the aerobic microorganisms in low-clay content soils but the aggravated hypoxia in high-clay content soils [40]. In contrast, N_2_O can be released, respectively, through nitrification under the aerobic environment and denitrification under the anaerobic environment. Therefore, soil aeration might be a decisive factor affecting not only N_2_O production but also the nitrogen content and the nitrogen availability of the soil organic matter. At the premise of climatic conditions during most of the year, the soil moisture should be more than 60% WFPS in JH but usually less than 60% in QG. Thus, to detect the difference of the variable N_2_O emission and CH_4_ uptake between the two research sites, the specific climatic conditions of two sites should also be individually taken into account.

### Regional GHG mitigation potential of *Miscanthus* production

Soil organic matter (SOM) plays a key role in improving soil quality and supporting vegetation growth, and is recognized as a main regulatory factor for GHGs [41]. Meanwhile, there is a close feedback effect between the released GHGs and regional climate system. Therefore, only the reductions in GHG fluxes (N_2_O, CH_4_, and CO_2_, mainly) could mitigate climate change. Aerobic soil mainly releases CO_2_ and N_2_O under normal climate conditions, but stimulates the reductions of N_2_O and CH_4_ productions and even hypoxia in the case of extreme weather (such as heavy rainfall in short term) [42]. In all, we needed to estimate and compare the magnitudes of the greenhouse gas release between the *Miscanthus* plantation and other types of vegetation in the region or elsewhere.

Compared with published field measurements, N_2_O and CH_4_ fluxes measured from *Miscanthus* fields in our study were in the same order of magnitude as annual average cumulative emission. The uptake rate of CH_4_-C was estimated from − 0.671 to 0.023 mg m^−2^ day^−1^ in our study, and the cumulative CH_4_ uptake indicated a net soil methane sink under most incubation conditions. In another report, the CH_4_ fluxes were ranging from − 0.386 to 0.452 g C m^−2^ day^−1^ reported from *Miscanthus* × *giganteus* in the arable soils of Lincolnshire, northeast England [43]. Moreover, the data of fluxes were ranging from − 0.070 to 0.014 g C m^−2^ day^−1^ according to another field experiment in Ihinger Hof, southwest Germany [9]. Compared with these studies, our release rates of CH_4_-C were within their ranges but below their average methane release. However, the release rate of N_2_O-N ranged from 0.015 to 0.154 mg m^−2^ day^−1^ in our study, which was much lower than reported ranging from 0.106 to 3.370 mg m^−2^ day^−1^ in a field experiment in southwest Germany [9], and also lower than another one ranging from − 0.264 to 6.600 mg m^−2^ day^−1^ in northeast England [43]. In our study, the results showed that *Miscanthus* plantation soil was a small net sink for atmospheric CH_4_ and a net source for N_2_O, estimated to be − 1.06 kg ha^−1^ year^−1^ CH_4_-C and 0.27 kg ha^−1^ year^−1^ N_2_O-N on average for the entire Loess Plateau region. In our study, N_2_O-N release in *Miscanthus* plantation was much less than in bare land (1.03 kg ha^−1^ year^−1^) and cropland of wheat (0.96 kg ha^−1^ year^−1^) [44, 45]. Meanwhile, the amount of CH_4_-C release in our estimated value is much less than the 5.60 kg ha^−1^ year^−1^ in wildwood and 0.94 kg ha^−1^ year^−1^ in wheat crop land [46]. The estimated annual regional average of N_2_O-N release was similar to the study of *Miscanthus sinensis*, which was at 0.07–0.36 kg ha^−1^ year^−1^ found in Kumamoto, Japan [46]. In addition to the amount of CO_2_ release [19], three major GHG emissions were 4.08 t CO_2eq_ ha^−1^ year^−1^ and the total GHG amount was 135.97 million t CO_2eq_ year^−1^ in the entire marginal lands available in the Loess Plateau (Fig. 5).

The soil nitrogen loss partly depends on the release of N_2_O under aerobic and anoxic environments [44]. Our research indicated that the process of soil N_2_O release was restrained in the *Miscanthus* plantation, which should be related to soil characteristics, the community composition and function of soil microorganism in the loess deposition region. Some interesting mechanisms must exist in this process, which will drive more attention in further research. The amount of nitrogen deposition shows a very low level in the region of Loess Plateau [47]. Therefore, the decrease of soil N_2_O emission could improve the utilization efficiency of soil nitrogen, and it will especially be more meaningful for the region with soil nitrogen deficiency.

### N_2_O emission of *Miscanthus* production and the utilization of soil nitrogen

There is a vast marginal land in the Loess Plateau, which could be an important area for bioenergy production. However, soil nitrogen could be a key-limiting factor in the energy plant growth. On the one hand, there was only a stingy amount of soil nitrogen fixation in the semi-arid area. On the other hand, nutrient loss in the top soil was relatively high because of the severe water and wind erosion [26]. Facing the N-limited environment, *Miscanthus* has several obvious advantages to adapt to marginal land, such as high nitrogen use efficiency [48], and as a perennial grass, the ability to internally cycle nutrients between aboveground tissues and rhizome. In addition, *Miscanthus* plants could reduce soil erosion and contribute to soil and water conservation because their dense and relatively shallow root systems can effectively adhere to soil and its large aboveground cover could prevent rain from scouring the surface of soil [19].

However, it seemed still difficult to support sustainable *Miscanthus* production in the marginal land without external fertilizer input because of the high nitrogen demands of high-yielding *Miscanthus* [49]. Moreover, ammonia volatilization in the weakly alkaline soil of loess regions caused a certain amount of soil nitrogen loss [50]. In this study, the content of soil total nitrogen decreased by 0.34 mg N g^−1^ in the 0–20 cm soil layer in QG from 2012 to 2014, which is equivalent to 425.00 kg ha^−1^ year^−1^ (Additional file 1: Fig. S4). Considering that the biomass of *Miscanthus* usually contains 1% nitrogen [51], the yield of *M. lutarioriparius* reached 2.90 × 10^4^ kg ha^−1^ year^−1^ in the QG site [32], which accounted for 68.24% (SE = 3.44%) of the total soil nitrogen reduction from 2012 to 2014. If soil nutrient reserve was seriously depleted, the yield of *Miscanthus* will be greatly affected and it was impossible for the yield to maintain at such a high level in the years of study. A possible explanation may be N fixation given that nitrogenase activity was found via acetylene reduction in rhizomes and in bacteria isolated from the rhizosphere [52].

In this study, we found that the annual release amount of N_2_O-N was 0.33 kg ha^−1^ year^−1^ in QG, which accounted for just 0.08% of the soil total nitrogen reduction in the period of 2012–2014. The proportion of nitrogen loss through N_2_O was much lower than that in other similar research regions [44–46]. Although our assessments did not cover the complete nitrogen budget, these results suggested that the amount of nitrogen lost largely supported the *Miscanthus* growth, while a very small percentage of nitrogen was released into the atmosphere as N_2_O. Taken together, we speculated that *Miscanthus* may adapt to the semi-arid marginal land with nitrogen limitation using a strategy of low N_2_O emission. A plausible explanation is that soil microbial activity limited N_2_O release from the bulk soil in the *Miscanthus* field. Specifically, the rhizosphere of *Miscanthus* might recruit some microbial communities, such as *Pseudomonas fluorescens*, which carried the N_2_O reductase gene and could dominate nitrite reductase in soil nitrogen turnover [53]. These complex interactions between microbial community and the root of *Miscanthus* need thorough evaluation in future studies.

## Conclusions

In summary, the work presented here offers a spatial estimate of the GHG emissions from *Miscanthus* energy crop production in the Loess Plateau of China. We developed a model to link the laboratory incubation conditions and regional climatic factors and could further estimate the potential N_2_O and CH_4_ emissions for the entire region. Considering the effects of N_2_O and CH_4_ on global warming converted to the equivalents of CO_2_, we found that the greenhouse effect of *Miscanthus* plantation was less than those of the crop land, grassland and shrubs. Although the Loess Plateau is one of the severe soil erosion regions with arid climate, poor soil nutrient and lack of vegetation, the study strengthened that *Miscanthus* energy crop would show great potential in reducing GHG emissions in the vast marginal lands across the infertile Loess Plateau.

## Methods

### Study sites and sampling

This study was conducted at the two *Miscanthus* sites established in 2009. One experimental field was located in Qingyang, Gansu province (QG) (35°42′N, 107°32′E), near the domestic habitat. The other was located in Jiangxia, Hubei province (JH) (30°21′N, 114°19′E), near the native habitat. QG is the loessial hilly gully area with semi-arid climate while JH is the subtropical humid plain. The mean annual precipitation ranges from 450 to 500 mm in QG and 1100 to 1200 mm in JH. The mean annual air temperature in QG and JH ranges from 7.0 to 10.0 °C and 15.8 to 17.5 °C, respectively. The soil type is loessial or dark loessial soil in QG and is yellow-brown loam or red loam soil in JH (Table 3).

**Table 3:** Soil properties in the two layers at each *Miscanthus* site

Sites	Soil layer (cm)	Soil organic carbon (mg g^−1^)	Total N (mg g^−1^)	C/N ratio	pH	Bulk density (g cm^−3^)
QG	0–10	10.23 ± 0.57	0.89 ± 0.08	11.73 ± 0.74	8.54 ± 0.05	1.20 ± 0.07
	10–20	7.73 ± 0.16	0.73 ± 0.02	10.67 ± 0.15	8.60 ± 0.04	1.32 ± 0.08
JH	0–10	8.23 ± 0.88	0.98 ± 0.10	8.51 ± 0.54	5.88 ± 0.03	1.38 ± 0.05
	10–20	5.91 ± 0.71	0.95 ± 0.09	6.33 ± 0.48	6.02 ± 0.05	1.41 ± 0.04

In this study, three soil cores were sampled randomly in 1 m × 1 m quadrats of *M. lutarioriparius* in QG and JH in August 2012. Soil cores (5 cm in diameter) were taken close to the tillers from the depths of 0–10 cm and 10–20 cm in each quadrat using a soil auger. The soil samples were brought back to the laboratory for further analyses. The three soil cores from each quadrat were mixed in situ as one composite sample. Rocks and plant fragments were removed by hand. Soil bulk density and soil moisture at each depth were obtained using a cutting-ring method in the sampled quadrate. The soil samples were again collected in the same quadrats to determine the changes in the total nitrogen of soil using the same methods in QG in August, 2014. Soil sampling and pretreatment methods follow the standard procedures of Roberson et al. [54], and *t* test was performed to compare the difference of the total nitrogen of soil in QG in 2012 and in 2014.

### Measurement of soil properties

The soil samples were taken to the laboratory and air-dried and sieved through a 2-mm screen. Fine roots in soil were picked by electrostatic adhesion. The gravimetric moisture content of sieved soil was determined as the weight difference before and after oven-drying at 105 °C for 24 h to constant weight. Soil pH was determined in water suspension (water:soil = 2.5:1) by a pH meter (7065 Kent, Cambridge, UK). Soil water-filled pore space (WFPS) was calculated from bulk density and volumetric soil moisture content and was used to represent the soil moisture condition in the process of soil incubation. Soil total carbon (TC) and total nitrogen (TN) contents were analyzed by CHON analyzer (Elementar VARIO EL III, Hanau, Germany). Inorganic carbon (IC) was evaluated using a Calcimeter (Eijkelkamp, Giesbeek, Netherlands). The content of soil organic carbon (SOC) was equal to TC minus IC.

### Laboratory incubation experiments

Five grams of sieved soil subsamples of each soil sample were placed in a 60-mL flask in triplicates and three empty flasks were set as blank treatments. Additional distilled water was sprayed into the flask until the soil mass was held at 30%, 60%, and 90% WFPS. The flasks with soil were pre-incubated at 25 °C in dark for 7 days to get close to the field soil condition. Then the flasks were covered with semipermeable membrane and incubated at four temperatures: 5 °C, 15 °C, 25 °C, and 35 °C. Soil moisture was checked by weighing each flask every day and sprayed distilled water to maintain soil moisture content.

N_2_O and CH_4_ releasing from the soil were, respectively, measured on days 1, 2, 3, 7, 14, 21, and 28. After incubation, the rates at which N_2_O and CH_4_ accumulated in the headspace of the flask were measured with a gas chromatograph (Agilent HP 7890 SERIES II, Santa Clara CA, USA). For each treatment, flasks were sealed with rubber stoppers with one inlet and one outlet pinhole. Ambient atmospheric air was poured into the flask for 3 min until the flask completely tanked up. After 3-h incubation, 5 mL of gas was extracted from the headspace by gastight syringes with a three-way stopcock. After the measurement was finished, the flask was covered with semipermeable membrane and sealed the gap between the semipermeable membrane and the flask with parafilm. The air in the flask was set to be connected to the outside and soils were incubated for later experiment. The concentrations of N_2_O and CH_4_ in each gas sample were immediately determined with N_2_ as a carrier gas [54].

### Data processing of soil GHG emission

The measured concentrations of N_2_O and CH_4_ from all treatment samples were converted to mass units and also needed to correct for incubation conditions using the application of the ideal gas law, with *Y*_*m*_ representing the calculated mass of N or C (μg L^−1^):

1 $$Y_{m} = \frac{{Y_{v} MP}}{RT},$$

where *Y*_*v*_ is the headspace concentration of N_2_O or CH_4_ (μL L^−1^) in the day of measurement (eightfold of the measured value from the 3-h incubation time during the day of sampling), *M* is duple molecular weight of nitrogen (14.00 μg μmol^−1^) for N_2_O or carbon (12.01 μg μmol^−1^) for CH_4_, *P* is barometric pressure (in atmospheres, e.g., 1 atm), *R* is the universal gas constant (0.082 L atm mol^−1^ K), and *T* is the incubation temperature plus 273.15.

The mass of N or C in daily N_2_O or CH_4_ flux from unit dry soil (μg g^−1^) was calculated as

2 $$Y_{F} = \frac{{Y_{m} V}}{W},$$

where *Y*_*m*_ is obtained from Eq. (1), *V* is headspace volume of the flask (L), and *W* is dry mass equivalent of soil in the flask (g).

Because N_2_O or CH_4_ flux was not measured every day, *Y*_*F*_ between sampling intervals was calculated as the average of *Y*_*F*_ from the two adjacent sampling points. The cumulative mass (*Y*_*t*_) of N or C in N_2_O or CH_4_ flux (mg kg^−1^) in each sampling day (day *t*) during the 28-day incubation period was calculated as

3 $$Y_{t} = \sum\limits_{n = 1}^{t} {Y_{F,n} } ,$$

where *Y*_*F,n*_ represents *Y*_*F*_ of day *n* (μg g^−1^).

The potentials of greenhouse gas (N_2_O) emission (*Y*_0_) (mg kg^−1^) were estimated based on the kinetics of greenhouse gas flux:

4 $$Y_{t} = \frac{{Y_{0} t}}{{t_{0} + t}},$$

where *Y*_*t*_ is obtained from Eq. (3) and *t*_0_ is the semi-decomposition time (the number of days needed for reaching 50% of *Y*_0_). Multivariate non-linear regression analysis was performed to obtain *Y*_0_ and *t*_0_ for each treatment with SigmaPlot 10.0 (SYSTAT Software Inc., San Jose CA, USA).

The potentials of greenhouse gas (CH_4_) emission (*Y*_0_) (mg kg^−1^) were estimated based on the kinetics of greenhouse gas flux:

5 $$Y_{t} = \frac{{Y_{0} }}{{1 + \left( {\frac{t}{{t_{0} }}} \right)^{k} }},$$

where *Y*_*t*_ is obtained from Eq. (3), *t*_0_ is the semi-uptake time (the number of days needed for reaching 50% of *Y*_0_), and *k* is the mineralization rate constant (dimensionless). Multivariate non-linear regression analysis was performed to obtain *Y*_0_ and *t*_0_ for each treatment with SigmaPlot 10.0 (SYSTAT Software Inc., San Jose CA, USA).

Some fixed factors (soil depth, temperature, and moisture) and the random factor (SOC) affected *Y*_*t*_. These factors were analyzed using residual maximum likelihood implemented in Generalized Linear Mixed Model (GLMM) of PROC MIXED SAS 9.2 (SAS Institute Inc., Cary NC, USA). Differences in the N_2_O or CH_4_ emission among the treatments were analyzed with three-way ANOVA and a posteriori Duncan test when significant (*P* < 0.05) with SPSS 23.0 (IBM SPSS Inc., Chicago IL, USA).

### Estimate of GHG emission in the Loess Plateau

Annual potential of each greenhouse gas emission in a given site of 76 meteorological stations across the whole Loess Plateau (g ha^−1^ year^−1^ for N_2_O and CH_4_) under specific soil temperature and moisture conditions was calculated as

6 $$Y_{r} = \sum\limits_{j}^{365} {Y_{h,j}^{s,k} (T_{h,j} ,M_{h,j}^{s,k} )} ,$$

where $Y_{h,j}^{s,k}$ represents the average rate of potential greenhouse gas flux for soil depth *k* of soil type *s* in the site *h* on the day *j*, which was calculated as *Y*_0_/2*t*_0_ from Eqs. (4 and 5) under conditions of *T*_*h,j*_ and $M_{h,j}^{s,k}$. *T*_*h,j*_ represents the temperature level in site *h* on day *j*. Daily temperature and precipitation in 76 meteorological stations covering the whole Loess Plateau were obtained from the *China Meteorological Data Sharing Service System* (http://data.cma.cn/). $M_{h,j}^{s,k}$ represents the soil moisture level for soil depth *k* of soil type *s* in site *h* on day *j*, which was evaluated by the effect of precipitation on soil moisture in a given site (*h*). The relationship between soil hydrothermal status and meteorological conditions in three soil types was determined by soil properties [55, 56], and the specific formulas were established in the previous model [19]. Soil fluxes of N_2_O and CH_4_ gases were converted into CO_2_ equivalents, according to their global warming potential (GWP) over the past 100 years’ horizon from IPCC [57]. The GWPs of N_2_O and CH_4_ were estimated by applying the numerical of 310- and 25-fold to CO_2_, respectively. Thus, the gross effect of GHGs was calculated as

7 $${\text{GE}}_{\text{GHGs}} = Y_{{{\text{CO}}_{ 2} }} + aY_{{{\text{N}}_{ 2} {\text{O}}}} + bY_{{{\text{CH}}_{ 4} }} ,$$

where *a* and *b* are the CO_2_ equivalents of soil fluxes of N_2_O and CH_4_ gases. $Y_{{{\text{CO}}_{ 2} }}$ was calculated using the data of soil CO_2_ fluxes based on our previous work [19]. The map of modeled CH_4_-C, N_2_O-N, and CO_2_-C emission and the gross effect of GHGs were generated in 1 km × 1 km grid of the Loess Plateau by spatial interpolations of ordinary kriging method. All statistical analyses and model calculations were conducted using R 3.3.1 (R Foundation for Statistical Computing, Vienna, Austria). All spatial analyses were conducted using ArcGIS 9.3 (ESRI Inc., Redlands, CA, USA).

## Additional file

**Additional file 1: Fig. S1.** The effects of soil temperature and moisture on cumulative N_2_O-N and CH_4_-C of 10–20 cm soil layer in the sites of QG and JH. **Fig. S2.** The cumulative N_2_O-N emission with days of incubation under different temperatures and moistures in the 0–10 cm and 10–20 cm soil layers. **Fig. S3.** The cumulative CH_4_-C uptake with days of incubation under different temperatures and moistures in the 0–10 cm and 10–20 cm soil layers. **Fig. S4.** The changes of soil total nitrogen content in the 0–10 cm and 10–20 cm soil layers of of *Miscanthus *plantation in QG from 2012 to 2014. **Table S1.** Kinetic analyses of cumulative N_2_O-N of soil samples from two layers in QG under different incubation conditions. **Table S2.** Potential N_2_O-N release rates for different types of soils from two depths in QG under different temperature and moisture conditions calculated from Eq. (6). **Table S3.** Kinetic analyses of cumulative CH_4_-C of soil samples from two layers in QG under different incubation conditions. **Table S4.** Potential CH_4_-C uptake rates for different types of soils from two depths in QG under different temperature and moisture conditions calculated from Eq. (6).

## Abbreviations

CH_4_: methane
GHG: greenhouse gas
GLMM: generalized linear mixed model
GWP: global warming potential
IC: inorganic carbon
JH: Jiangxia of the Hubei Province in China
N_2_O: nitrous oxide
QG: Qingyang of the Gansu Province in China
SOC: soil organic carbon
SOM: soil organic matter
TC: soil total carbon
TN: soil total nitrogen
WFPS: soil water-filled pore space
