# Supplementary material for: N2O and CH4 emission from Miscanthus energy crop fields in the infertile Loess Plateau of China
Source: Biotechnol Biofuels. 2018 Dec 3;11:321. doi: 10.1186/s13068-018-1320-8 (PMC6276234; doi:10.1186/s13068-018-1320-8)
Supplement: Supplementary file 1 — Additional file 1: Fig. S1. The effects of soil temperature and moisture on cumulative N2O-N and CH4-C of 10–20 cm soil layer in the sites of QG and JH. Fig. S2. The cumulative N2O-N emission with days of incubation under different temperatures and moistures in the 0–10 cm and 10–20 cm soil layers. Fig. S3. The cumulative CH4-C uptake with days of incubation under different temperatures and moistures in the 0–10 cm and 10–20 cm soil layers. Fig. S4. The changes of soil total nitrogen content in the 0–10 cm and 10–20 cm soil layers of of Miscanthus plantation in QG from 2012 to 2014. Table S1. Kinetic analyses of cumulative N2O-N of soil samples from two layers in QG under different incubation conditions. Table S2. Potential N2O-N release rates for different types of soils from two depths in QG under different temperature and moisture conditions calculated from Eq. (6). Table S3. Kinetic analyses of cumulative CH4-C of soil samples from two layers in QG under different incubation conditions. Table S4. Potential CH4-C uptake rates for different types of soils from two depths in QG under different temperature and moisture conditions calculated from Eq. (6). [file 13068_2018_1320_MOESM1_ESM.doc]

**N_2_O and CH_4_ emission from *Miscanthus* energy crop fields in the infertile Loess Plateau of China**

Jia Mi^1,2,†^, Wei Liu^1,†^,*, Xuhong Zhao^1,3^, Lifang Kang^1^, Cong Lin^1^, Juan Yan^4^, and Tao Sang^1,5^,*

^1^Key Laboratory of Plant Resources and Beijing Botanical Garden, Institute of Botany, Chinese Academy of Sciences, Beijing 100093, China

^2^Institute of Loess Plateau and Shanxi Green Development Research Centre, Shanxi University, Taiyuan, Shanxi 030006, China

^3^University of Chinese Academy of Sciences, Beijing 100049, China

^4^Key Laboratory of Plant Germplasm Enhancement and Speciality Agriculture, Wuhan Botanical Garden, Chinese Academy of Sciences, Wuhan, Hubei 430074, China

^5^State Key Laboratory of Systematic and Evolutionary Botany, Institute of Botany, Chinese Academy of Sciences, Beijing 100093, China

^†^Jia Mi and Wei Liu contributed equally to this work.

*Correspondence: [sang@ibcas.ac.cn](mailto:sang@ibcas.ac.cn), liuw@ibcas.ac.cn

Jia Mi: ^1^Key Laboratory of Plant Resources and Beijing Botanical Garden, Institute of Botany, Chinese Academy of Sciences, Beijing 100093, China; ^2^Institute of Loess Plateau and Shanxi Green Development Research Centre, Shanxi University, Taiyuan, Shanxi 030006, China, jiami@sxu.edu.cn

Wei Liu: ^1^Key Laboratory of Plant Resources and Beijing Botanical Garden, Institute of Botany, Chinese Academy of Sciences, Beijing 100093, China, [liuw@ibcas.ac.cn](mailto:liuw@ibcas.ac.cn)

Xuhong Zhao: ^1^Key Laboratory of Plant Resources and Beijing Botanical Garden, Institute of Botany, Chinese Academy of Sciences, Beijing 100093, China; ^3^University of Chinese Academy of Sciences, Beijing 100049, China, **zhaoxh17@ibcas.ac.cn**

Lifang Kang: ^1^Key Laboratory of Plant Resources and Beijing Botanical Garden, Institute of Botany, Chinese Academy of Sciences, Beijing 100093, China,

**kanglf@ibcas.ac.cn**

Cong Lin: ^1^Key Laboratory of Plant Resources and Beijing Botanical Garden, Institute of Botany, Chinese Academy of Sciences, Beijing 100093, China,

**lincong@ibcas.cn**

Juan Yan: ^4^Key Laboratory of Plant Germplasm Enhancement and Speciality Agriculture, Wuhan Botanical Garden, Chinese Academy of Sciences, Wuhan, Hubei 430074, China, **yanj@wbgcas.cn**

Tao Sang: ^1^Key Laboratory of Plant Resources and Beijing Botanical Garden, Institute of Botany, Chinese Academy of Sciences, Beijing 100093, China; ^5^State Key Laboratory of Systematic and Evolutionary Botany, Institute of Botany, Chinese Academy of Sciences, Beijing 100093, China, [sang@ibcas.ac.cn](mailto:sang@ibcas.ac.cn)

The full postal address: No.20 Nanxincun, Xiangshan, Beijing 100093, China


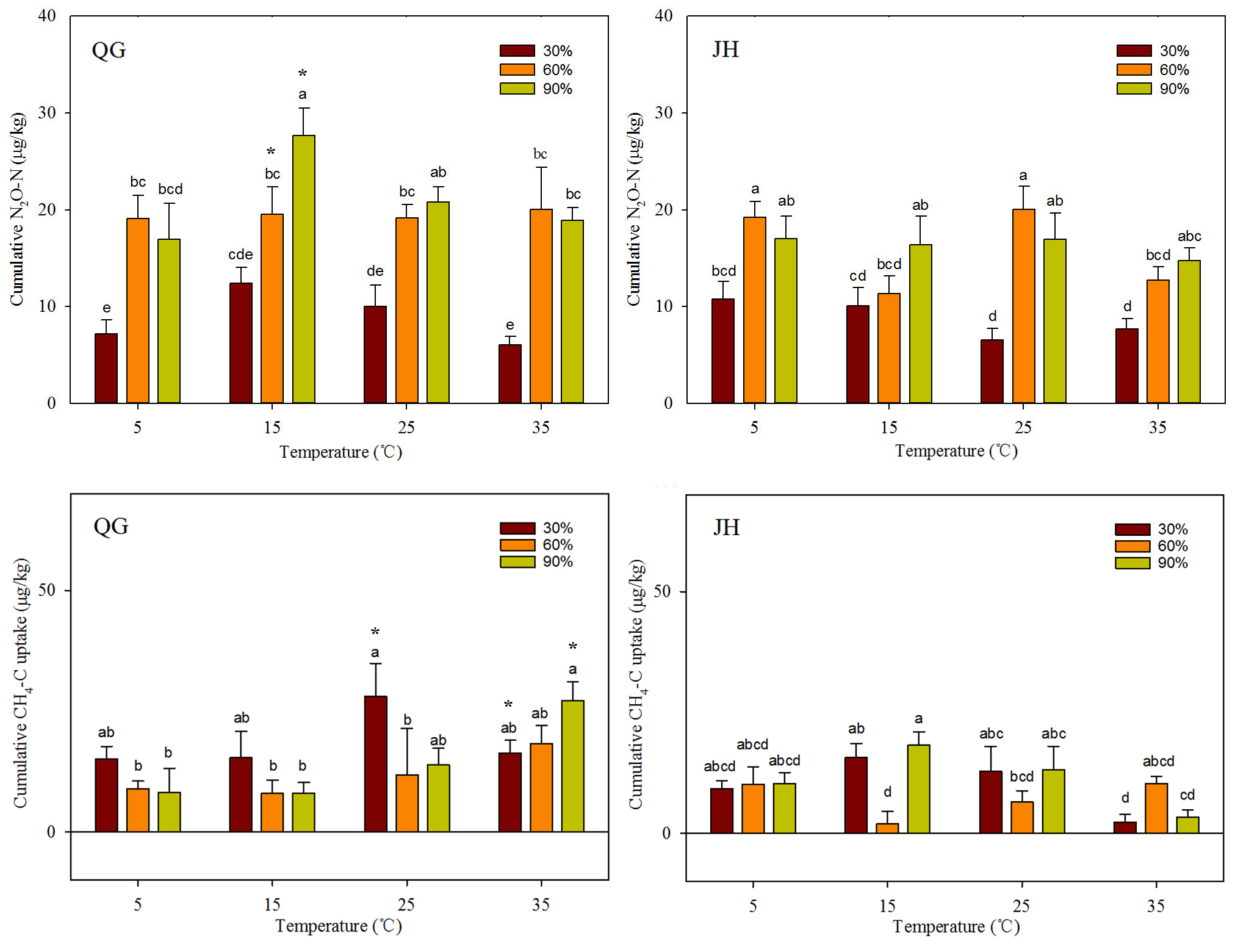


**Fig. S1** The effects of soil temperature and moisture on cumulative N_2_O-N and CH_4_-C emissions of 10-20cm soil layer in the sites of QG and JH. The cumulative N_2_O-N and CH_4_-C for each treatment was the average of three replicates (error bars denote SEM). Bars with the same letter were not significantly different in the least significant difference (LSD) tests reported from ANOVA. * indicates a significant level of difference between QG and JH under same soil temperature and moisture conditions at *P* = 0.05.


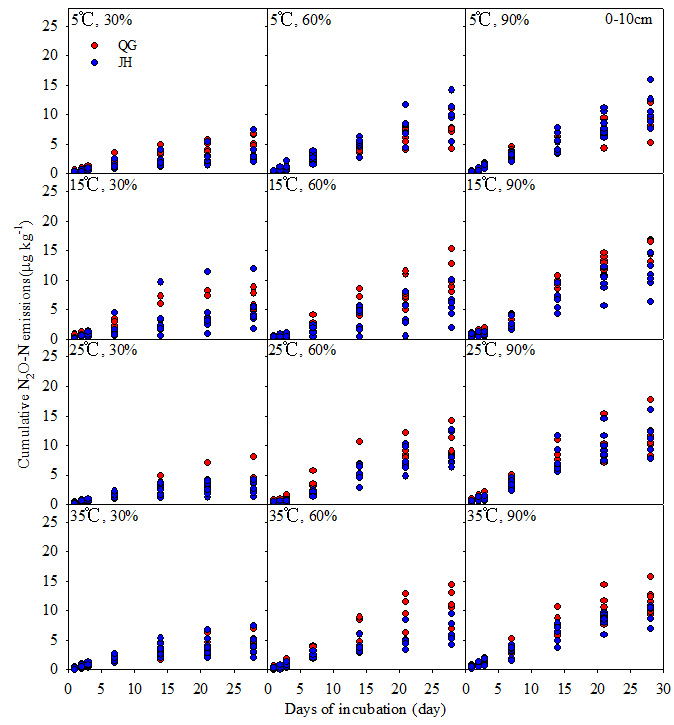

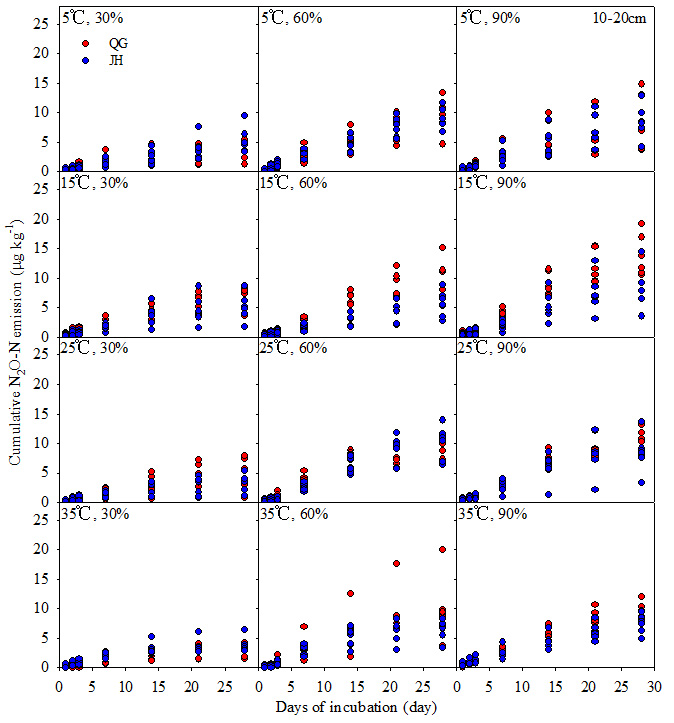


**Fig. S2** The cumulative N_2_O-N emission with days of incubation under different temperatures and moistures in the 0-10 cm and 10-20 cm soil layers.


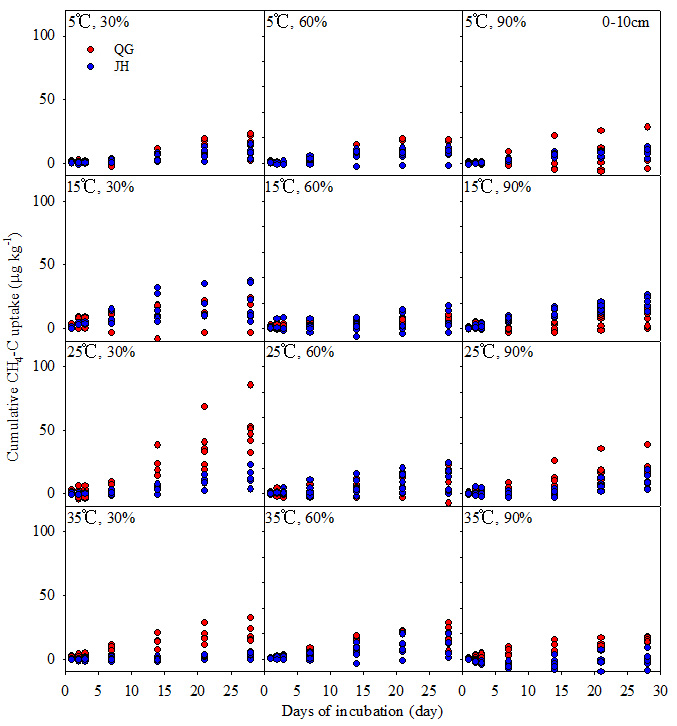

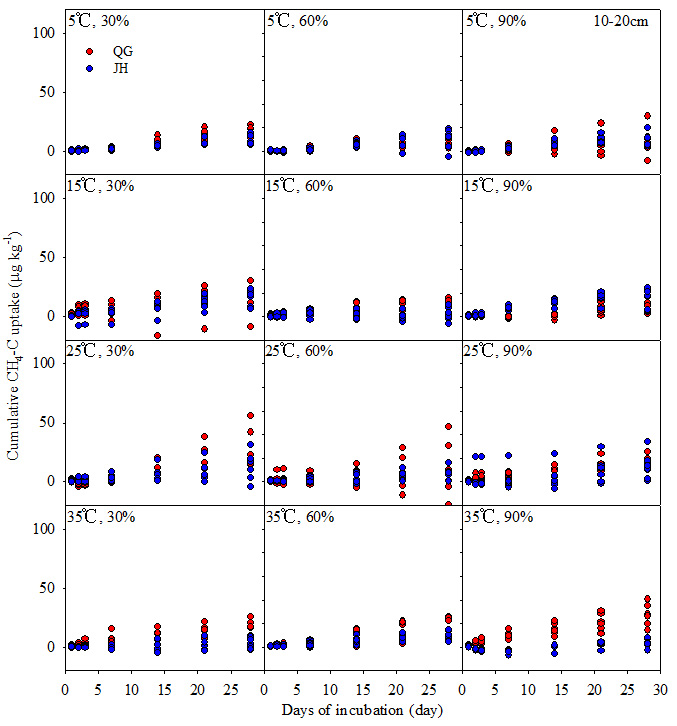


**Fig. S3** The cumulative CH_4_-C uptake with days of incubation under different temperatures and moistures in the 0-10 cm and 10-20 cm soil layers.


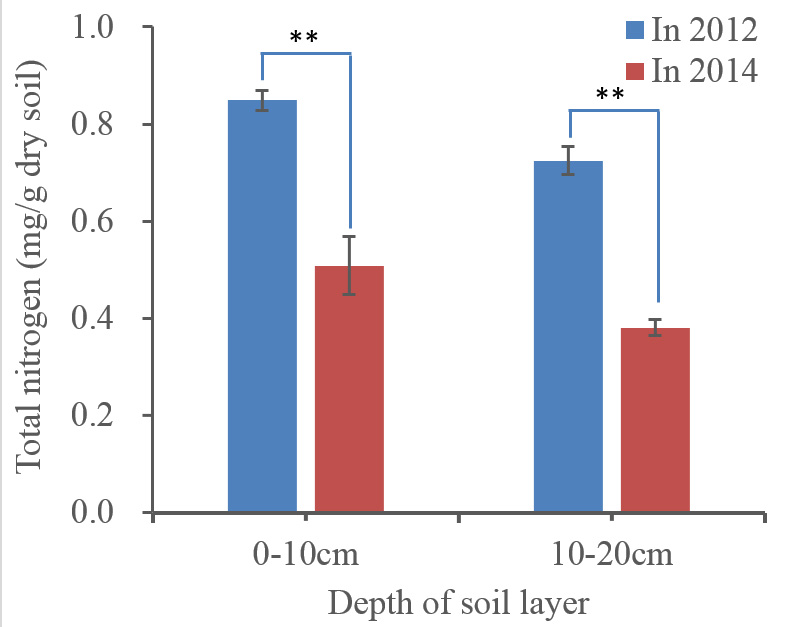


**Fig. S4** The changes of soil total nitrogen content in the 0-10 cm and 10-20 cm soil layers of *Miscanthus* plantation in QG from 2012 to 2014. ** indicates a significant level of difference at *P* = 0.01.

**Table S1 Kinetic analyses of cumulative N_2_O-N of soil samples from two layers in QG under different incubation conditions.**

| Soil depth  (cm) | Temperature  (°C) | Moisture  (% WFPS) | *Nt*=*N_0_t*/*t_0_*+*t* | | | |
| --- | --- | --- | --- | --- | --- | --- |
|  |  |  | R^2^ | *P* | *N*_0_ | *t*_0_ |
| 0–10 | 5 | 30 | 0.74 | <0.0001 | 15.06 | 35.70 |
|  | 5 | 60 | 0.88 | <0.0001 | 89.82 | 257.90 |
|  | 5 | 90 | 0.86 | <0.0001 | 49.18 | 95.42 |
|  | 15 | 30 | 0.69 | <0.0001 | 26.66 | 69.24 |
|  | 15 | 60 | 0.84 | <0.0001 | 194.82 | 472.30 |
|  | 15 | 90 | 0.97 | <0.0001 | 196.70 | 286.66 |
|  | 25 | 30 | 0.77 | <0.0001 | 22.14 | 72.74 |
|  | 25 | 60 | 0.87 | <0.0001 | 58.36 | 104.2 |
|  | 25 | 90 | 0.83 | <0.0001 | 55.44 | 81.12 |
|  | 35 | 30 | 0.81 | <0.0001 | 20.92 | 72.86 |
|  | 35 | 60 | 0.80 | <0.0001 | 115.60 | 250.22 |
|  | 35 | 90 | 0.91 | <0.0001 | 80.96 | 129.08 |
| 10–20 | 5 | 30 | 0.55 | <0.0001 | 10.18 | 27.82 |
|  | 5 | 60 | 0.83 | <0.0001 | 193.36 | 504.46 |
|  | 5 | 90 | 0.61 | <0.0001 | 37.06 | 68.50 |
|  | 15 | 30 | 0.80 | <0.0001 | 23.74 | 49.44 |
|  | 15 | 60 | 0.82 | <0.0001 | 90.82 | 194.80 |
|  | 15 | 90 | 0.89 | <0.0001 | 105.70 | 151.44 |
|  | 25 | 30 | 0.62 | <0.0001 | 25.34 | 82.92 |
|  | 25 | 60 | 0.91 | <0.0001 | 47.82 | 78.52 |
|  | 25 | 90 | 0.93 | <0.0001 | 68.80 | 121.56 |
|  | 35 | 30 | 0.72 | <0.0001 | 11.38 | 46.82 |
|  | 35 | 60 | 0.63 | <0.0001 | 76.66 | 151.62 |
|  | 35 | 90 | 0.93 | <0.0001 | 69.42 | 143.3 |

**Notes:** WFPS is the abbreviation of soil water filled pore space; *N_t_* is the cumulative mass of N in N_2_O in each sampling day during the 28-day incubation period; *N_0_* is the potentials of greenhouse gases (N_2_O) emission; *t_0_* is semi-decomposition time (the number of days needed for reaching 50% of *N_0_*; *t* is the sampling day.

**Table S2 Potential N_2_O-N release rates for different types of soils from two depths in QG under different temperature and moisture conditions calculated from equation (6).**

| Soil types | Depth |  | N_2_O-N (g ha^-1^ day^-1^) | | |
| --- | --- | --- | --- | --- | --- |
|  |  |  | 30% | 60% | 90% |
| Dark loessial soil | 0–10 cm | <5°C | 0.16 | 0.18 | 0.34 |
|  |  | 5°C | 0.54 | 0.46 | 0.68 |
|  |  | 15°C | 0.50 | 0.54 | 0.90 |
|  |  | 25°C | 0.40 | 0.72 | 0.88 |
|  |  | 35°C | 0.38 | 0.60 | 0.82 |
|  |  |  |  |  |  |
|  | 10–20 cm | <5°C | 0.20 | 0.28 | 0.48 |
|  |  | 5°C | 0.52 | 0.54 | 0.76 |
|  |  | 15°C | 0.68 | 0.66 | 0.98 |
|  |  | 25°C | 0.42 | 0.86 | 0.80 |
|  |  | 35°C | 0.34 | 0.70 | 0.68 |
| Yellow cultivated loessial soil | 0–10 cm | <5°C | 0.16 | 0.18 | 0.32 |
|  |  | 5°C | 0.50 | 0.42 | 0.62 |
|  |  | 15°C | 0.46 | 0.50 | 0.82 |
|  |  | 25°C | 0.36 | 0.68 | 0.82 |
|  |  | 35°C | 0.34 | 0.56 | 0.76 |
|  |  |  |  |  |  |
|  | 10–20 cm | <5°C | 0.20 | 0.26 | 0.46 |
|  |  | 5°C | 0.48 | 0.50 | 0.70 |
|  |  | 15°C | 0.62 | 0.60 | 0.90 |
|  |  | 25°C | 0.40 | 0.80 | 0.74 |
|  |  | 35°C | 0.32 | 0.66 | 0.62 |
| Aeolian sandy soils | 0–10 cm | <5°C | 0.10 | 0.12 | 0.20 |
|  |  | 5°C | 0.34 | 0.28 | 0.42 |
|  |  | 15°C | 0.30 | 0.32 | 0.54 |
|  |  | 25°C | 0.24 | 0.44 | 0.54 |
|  |  | 35°C | 0.22 | 0.36 | 0.50 |
|  |  |  |  |  |  |
|  | 10–20 cm | <5°C | 0.12 | 0.16 | 0.28 |
|  |  | 5°C | 0.30 | 0.30 | 0.44 |
|  |  | 15°C | 0.38 | 0.38 | 0.56 |
|  |  | 25°C | 0.24 | 0.48 | 0.46 |
|  |  | 35°C | 0.20 | 0.40 | 0.38 |

**Table S3 Kinetic analyses of cumulative CH_4_-C of soil samples from two layers in QG under different incubation conditions.**

| Soil depth  (cm) | Temperature  (°C) | Moisture  (% WFPS) | *C’t = C’_0_/(1+(t/t_0_)^k^)* | | | |
| --- | --- | --- | --- | --- | --- | --- |
|  |  |  | R^2^ | *P* | *C’*_0_ | *t*_0_ |
| 0–10 | 5 | 30 | 0.87 | <0.0001 | 13.48 | 13.38 |
|  | 5 | 60 | 0.99 | <0.0001 | 43.48 | 73.01 |
|  | 5 | 90 | 0.98 | <0.0001 | 4.77 | 1.87 |
|  | 15 | 30 | 0.95 | <0.0001 | 17.43 | 7.68 |
|  | 15 | 60 | 0.99 | <0.0001 | 8.89 | 10.09 |
|  | 15 | 90 | 0.99 | <0.0001 | 7.19 | 18.98 |
|  | 25 | 30 | 0.89 | <0.0001 | 80.80 | 22.60 |
|  | 25 | 60 | 0.95 | <0.0001 | 17.42 | 19.29 |
|  | 25 | 90 | 0.96 | <0.0001 | 13.86 | 4.60 |
|  | 35 | 30 | 0.92 | <0.0001 | 14.86 | 2.14 |
|  | 35 | 60 | 0.95 | <0.0001 | 27.25 | 17.02 |
|  | 35 | 90 | 0.99 | <0.0001 | 9.52 | 1.71 |
| 10–20 | 5 | 30 | 0.89 | <0.0001 | 15.92 | 12.89 |
|  | 5 | 60 | 0.99 | <0.0001 | 0.11 | 0.08 |
|  | 5 | 90 | 0.97 | <0.0001 | 4.93 | 3.41 |
|  | 15 | 30 | 0.93 | 0.0433 | 10.83 | 3.12 |
|  | 15 | 60 | 0.99 | 0.0005 | 10.44 | 9.34 |
|  | 15 | 90 | 0.99 | <0.0001 | 8.69 | 18.38 |
|  | 25 | 30 | 0.93 | <0.0001 | 40.25 | 20.80 |
|  | 25 | 60 | 0.89 | 0.0082 | 46.28 | 15.99 |
|  | 25 | 90 | 0.98 | 0.0428 | 6.55 | 30.44 |
|  | 35 | 30 | 0.97 | <0.0001 | 11.40 | 1.19 |
|  | 35 | 60 | 0.98 | <0.0001 | 32.61 | 23.71 |
|  | 35 | 90 | 0.96 | <0.0001 | 86.45 | 50.08 |

**Notes:** WFPS is the abbreviation of soil water filled pore space; *C’_t_* is the cumulative mass of C in CH_4_ in each sampling day during the 28-day incubation period; *C’_0_* is the potentials of greenhouse gases (CH_4_) emission; *t_0_* is semi-decomposition time (the number of days needed for reaching 50% of *C’_0_*; *t* is the sampling day; *k* is the soil organic carbon converting to methane rate constant (dimensionless).

**Table S4 Potential CH_4_-C uptake rates for different types of soils from two depths in QG under different temperature and moisture conditions calculated from equation (6).**

| Soil types | Depth |  | CH_4_-C (g ha^-1^ day^-1^) | | |
| --- | --- | --- | --- | --- | --- |
|  |  |  | 30% | 60% | 90% |
| Dark loessial soil | 0–10 cm | <5°C | 0.85 | 0.49 | 1.91 |
|  |  | 5°C | 0.65 | 0.39 | 1.28 |
|  |  | 15°C | 1.48 | 0.57 | 0.19 |
|  |  | 25°C | 2.32 | 0.59 | 1.51 |
|  |  | 35°C | 4.51 | 1.04 | 2.78 |
|  |  |  |  |  |  |
|  | 10–20 cm | <5°C | 3.46 | -0.23 | 2.75 |
|  |  | 5°C | 0.86 | 0.96 | 1.01 |
|  |  | 15°C | 2.43 | 0.78 | 0.33 |
|  |  | 25°C | 1.35 | 2.03 | 0.15 |
|  |  | 35°C | 6.71 | 0.96 | 1.21 |
| Yellow cultivated loessial soil | 0–10 cm | <5°C | 0.78 | 0.45 | 1.76 |
|  |  | 5°C | 0.60 | 0.36 | 3.18 |
|  |  | 15°C | 1.36 | 0.53 | 4.27 |
|  |  | 25°C | 2.15 | 0.54 | 2.92 |
|  |  | 35°C | 4.17 | 0.96 | 1.95 |
|  |  |  |  |  |  |
|  | 10–20 cm | <5°C | 3.21 | -0.22 | 2.55 |
|  |  | 5°C | 0.80 | 0.89 | 0.94 |
|  |  | 15°C | 2.26 | 0.73 | 0.31 |
|  |  | 25°C | 1.26 | 1.88 | 0.14 |
|  |  | 35°C | 6.23 | 0.89 | 1.12 |
| Aeolian sandy soils | 0–10 cm | <5°C | 0.52 | 0.30 | 1.17 |
|  |  | 5°C | 0.40 | 0.24 | 2.12 |
|  |  | 15°C | 0.91 | 0.35 | 2.84 |
|  |  | 25°C | 1.43 | 0.36 | 1.95 |
|  |  | 35°C | 2.78 | 0.64 | 1.30 |
|  |  |  |  |  |  |
|  | 10–20 cm | <5°C | 1.98 | -0.13 | 1.57 |
|  |  | 5°C | 0.49 | 0.55 | 0.58 |
|  |  | 15°C | 1.39 | 0.45 | 0.19 |
|  |  | 25°C | 0.77 | 1.16 | 0.09 |
|  |  | 35°C | 3.83 | 0.55 | 0.69 |
